# Editorial: Emerging molecular mechanisms in cardiovascular physiology and pathology

**DOI:** 10.3389/fphys.2024.1485595

**Published:** 2024-09-10

**Authors:** Junco S. Warren, Daniel M. Johnson, Francesco Paneni, Prabhu Mathiyalagan

**Affiliations:** ^1^ Virginia Tech Carilion, Roanoke, VA, United States; ^2^ School of Life, Health and Chemical Sciences, The Open University, Milton Keynes, United Kingdom; ^3^ Department of Cardiology, University Heart Center, University Hospital Zurich, Zurich, Switzerland; ^4^ Department of Cardiology, Center for Translational and Experimental Cardiology (CTEC), Zurich University Hospital, University of Zurich, Schlieren, Switzerland; ^5^ Benthos Prime Central, Houston, TX, United States

**Keywords:** cardiovascular, metabolism, endothelium, cardiac fibrosis, microRNA

## Introduction

The cardiovascular system is meticulously regulated by the intricate interplay between distinct cell types, with multi-level cellular crosstalk being crucial for maintaining homeostasis. Disruptions at the molecular level, including genetic and epigenetic modifications, cellular dysfunction, impaired immune cell function and lipidome, can lead to cardiovascular pathology (Mathiyalagan et al., 2019; Costantino et al., 2023; Warren et al., 2018). Traditionally, research has focused on key cell types such as cardiomyocytes, fibroblasts, endothelial cells, and smooth muscle cells (Deckx et al., 2019). However, recent advances in single-cell transcriptomics sequencing have revealed greater cellular diversity within the heart and its vascular microenvironment, highlighting the pathological roles of fibroblasts, various immune cells and their distinct subtypes under normal physiological and pathological states (Miranda et al., 2023). The complex interactions between these diverse cell populations are now recognized as critical mediators of pathological signaling. While targeting specific cell types, including immune cells, endothelial cells, and fibroblasts, shows clinical promise, the precise molecular mechanisms and communication pathways among these cells remain incompletely understood.

This RNA Topic, titled “*Emerging Molecular Mechanisms in Cardiovascular Physiology and Pathology*” highlights the importance of intricate cellular crosstalk within the cardiovascular system. The articles in this topic explore miRNA mechanisms, lipid metabolism, cardiac fibroblasts, endothelial cells, cardiomyocyte crosstalk, and left ventricular function in astronauts on space missions. A total of six articles have been published in this RNA Topic, including four original articles and two review articles.

In the original research article, Ainiwan et al. describe stem cell transplantation for the treatment of acute myocardial infarction (AMI), with a particular focus on the microRNA-1 (miRNA-1) mediated regulatory mechanisms within the myocardial microenvironment post-stem cell transplantation. They address the challenge of delivering naked miRNA-1, which is extremely unstable, non-targeted, and rapidly degraded by circulating RNase. To overcome this challenge, the authors explore the construction of pseudo-endogenous miRNA-1 targeted myocardial ultrasound nanobubbles (NB) pAd-AAV-9/miRNA-1 NB and evaluate their characteristics, targeting, and function. By linking the pAd-AAV-9/miRNA-1 gene complex to NB using an “avidin-biotin bridging” method, they target cardiomyocytes. The study demonstrates that NB particle size and concentration remain stable after delivery, with good contrast-enhanced ultrasound imaging observed *in vivo*. The results show that miRNA-1 expression in the heart of the rats increased over time while decreasing in other vital organs, with consistently higher levels in the heart. The overexpression of pAd-AAV-9/miRNA-1 NB exhibited cardiac specificity, accumulating primarily in myocardial tissue. Although some initial accumulation was observed in the lungs, it rapidly decreased after 15 min, consistent with the metabolism of lipid ultrasound nanobubbles through pulmonary circulation.

Brojakowska et al. address a critical challenge faced by astronauts on prolonged space missions, where they are exposed to space radiation (IR), which increases the risk of IR-induced cardiovascular disease. To study the effects of space radiation on left ventricular (LV) function, they used Apolipoprotein E (ApoE) null mice irradiated with gamma radiation (IR) and assessed LV function using transthoracic echocardiography at various time points post-irradiation. At days 14 and 28 post-irradiation, they observed a decrease in LV systolic function across all IR doses. At later stages, a significant decrease in LV systolic function was noted. To gain molecular insights, the authors evaluated the expression of genes involved in hemodynamic stress, cardiac remodeling, inflammation, and calcium handling in LVs harvested from ApoE null mice 28 days post-IR. They found increased expression of *Bnp* and *Ncx* genes, suggesting impaired hemodynamic stress and altered calcium handling. Furthermore, *Gals3* and *β-Mhc* levels were elevated, indicating cardiac remodeling events.

Lipid metabolism plays a critical role in endothelial cell (EC) function and tissue homeostasis. Bousquet et al. describe how liver X receptors (LXRs) are involved in the transcriptional regulation of genes associated with EC function and how LXRs can be targeted for cardiovascular disease treatment. To study the effects of LXRs in ECs, the authors exposed ECs to the LXR agonist T0901317 and demonstrated that LXR activation increased polyunsaturated fatty acids (PUFAs) and decreased saturated fatty acids, as well as reduced the uptake of fatty acids (FAs) by ECs. The authors showed that this effect was mediated by LXRalpha, as silencing LXRalpha abolished the effects of the LXR agonist in ECs. Transcriptomic analysis using PCR identified increased expression of lysophosphatidylcholine acyltransferase (LPCAT3), which is involved in the turnover of FAs. Furthermore, LXR agonist increased the expression of key genes involved in the synthesis of PUFAs, including FA desaturase 1 and 2, FA elongase 5, and fatty acid synthase. Interestingly, the authors identified a global impact on lipid metabolism in ECs exposed to the LXR agonist, including changes in arachidonic acid, eicosapentaenoic acid, and docosahexaenoic acid content in ECs.

Marrow et al. examined the paracrine effects of erythropoietin (EPO) in cardiomyocyte-endothelial cell crosstalk. The authors studied cardiomyocyte-specific EPO knockout mice and showed that the loss of EPO in cardiomyocytes led to compensatory overexpression of EPO in the heart by endothelial cells. At the physiological level, the EPO knockout mice demonstrated greater voluntary wheel running capacity than control mice. The authors found that the cells of the LV wall had widened, the length of the hearts had shortened, but there was no difference in global heart mass. By ruling out pathological hypertrophy based on no observed differences in certain fetal gene re-activation, the authors identified concentric cellular hypertrophy with no evidence of cardiac fibrosis in EPO knockout mice. Furthermore, the authors observed heightened cardiac function *in vivo* in EPO knockout mice. At the molecular level, they provided evidence of elevated Vegfr1 and Vegfb RNA expression, which accompanied the hyper-compensated endothelial EPO expression, and this was further modulated upon pharmacological pan-inhibition of VEGF-VEGFR signaling. Together, the authors discovered a new mechanism where the deletion of EPO in adult cardiomyocytes triggers endothelial cell-derived EPO and subsequent Vegfb expression, promoting cardiomyocyte hypertrophy in a feedforward loop.

In the review article, Hoque et al. focus on cardiac fibrogenesis from an immune-metabolic perspective. They discuss how the cardiac non-myocyte population, such as fibroblasts, undergoes adaptive changes, immune cell transformations, and metabolic shifts. These changes initially offer protection during pathological events but eventually lead to adverse remodeling through altered energy metabolism, mitochondrial dysfunction, and interactions between immune cells and cardiomyocytes. These processes drive fibroblast transdifferentiation and remodeling. Targeting metabolic plasticity, fibroblast-to-myofibroblast transition, and immune response modulation could help manage fibrosis in cardiovascular disease. In this context, the authors comprehensively review the literature and highlight the significance of the immune system’s contribution to cardiac homeostasis, the emerging roles of immune cells in cardiac fibrosis, and the therapeutic targeting of immune cells to manage cardiac fibrosis. Moreover, the authors describe how metabolic regulation governs cardiac homeostasis, the impact of metabolic reprogramming in the stressed heart, and how metabolism dysregulation contributes to cardiac fibrosis. Interestingly, the article explores how immune cell metabolism may play a role in cardiac fibrogenesis, underscoring the interplay between the immune system and metabolism in cardiac fibrosis and how this interplay could be targeted therapeutically to prevent or cure cardiac fibrosis.

Jiang et al. review the critical involvement of the developmental endothelial locus 1 (DEL-1 or EDIL3) gene in immunoregulation and vascular biology. DEL-1, which is produced by endothelial cells (ECs), regulates immune cells through direct interactions with integrins, preventing unnecessary immune cell recruitment and inflammation. The authors discuss how DEL-1 could serve as a potential therapeutic target in immune-mediated blood disorders, cardiovascular disease, and cancer metastasis, given its integral role in vascular integrity and pathology. They describe the mechanisms by which DEL-1 interacts with T-cells, macrophages, and platelets. Furthermore, the role of DEL-1 in atherosclerosis, angiogenesis, and endoplasmic reticulum stress is also discussed. The authors propose a clear model of how DEL-1 could be a potential therapeutic target or even a biomarker for vascular diseases.
